# The relationship of academic procrastination on non-English majors’ English classroom anxiety: a moderated mediation model

**DOI:** 10.3389/fpsyg.2024.1391779

**Published:** 2024-09-16

**Authors:** Feng Jiang

**Affiliations:** School of Language, Nanjing Normal University of Special Education, Nanjing, China

**Keywords:** non-English majors, academic procrastination, English classroom anxiety, learning engagement, appraisals of intrinsic values

## Abstract

**Objective:**

Academic procrastination is negatively associated with English classroom anxiety among non-English major college students. However, current research has less explored the underlying mechanisms. The present study aims to investigate the relationship between academic procrastination and college students’ English classroom anxiety as well as the mediating role of learning engagement and the moderating role of appraisals of intrinsic values.

**Methods:**

The academic procrastination scale, English classroom anxiety scale, learning engagement scale and appraisals of intrinsic values scale were used to measure 1,079 non-English majors in Jiangsu Province, China.

**Results:**

(1) There was a significant positive correlation between academic procrastination and English classroom anxiety of non-English majors. (2) Learning engagement plays a part of mediating role between academic procrastination and English classroom anxiety; (3) When the appraisals of intrinsic values are high, the impact effect of learning engagement on English classroom anxiety is greater.

**Conclusion:**

Academic procrastination can affect university students’ English classroom anxiety through learning engagement, and this relational pattern is modulated by appraisals of intrinsic values. This finding provides an important theoretical basis and practical insights for understanding and intervening in academic procrastination and classroom anxiety among college students.

## 1 Introduction

The importance of English in today’s world is indisputable; not only is it the most widely spoken second language globally, but it has also become an almost universal bridge for international communication, academic co-operation and business dealings (Melitz, 2016). In China’s education system, English is also taught as a compulsory subject in primary to tertiary education. One study found that 93.8% of China’s 416 million foreign language learners study English (Wei and Su, 2012). For Chinese non-English speaking college students, mastering English is not only a way to cope with academic exams, but also a way to enhance one’s employment opportunities (Bolton and Graddol, 2012). However, many Chinese non-English major college students often suffer from anxiety when taking English classes. These negative affective factors not only affect their learning efficiency, but may also adversely affect their future career development. Therefore, it is of great practical significance to explore the relationship between academic procrastination and anxiety to avoid the emergence of anxiety among non-English major college students.

### 1.1 Academic procrastination and English classroom anxiety

Academic procrastination can be understood as the realization that one should complete an academic task, but fails to do so within the desired time frame (Steel, 2007; Wolters, 2003). As a kind of poor learning behavior and habit, academic procrastination is prevalent among university students, and is one of the main manifestations of academic problems, which greatly hinders the improvement of students’ learning enthusiasm and academic record (Çapan, 2010). Research has found that procrastination reduces learners’ confidence and lowers their expectation of completing tasks, and then causes anxiety and negatively affects learners’ achievement of objectives (Wang, 2021). Anxiety in language learning mainly refers to the fear or unease of learners when they need to express themselves in a foreign language or a second language, and belongs to the anxiety response in a specific environment (Gardner and MacIntyre, 1993). In university English teaching, learning anxiety is widely present in classroom activities throughout the entire English learning process, and is gradually becoming an emotional factor that has attracted much attention (Woodrow, 2011), which directly or indirectly affects students’ academic level. It has been found that when English learners are afraid of classroom learning, they are often manifested by too much care about teachers’ or peers’ evaluation of their English abilities (Azher et al., 2010; Matsuda and Gobel, 2004). Therefore, when university students face English learning tasks, they may feel nervous and worried that they are not performing as well as others or unable to meet expected standards, and thus are reluctant to actively participate in the classroom activities. Academic procrastination also often leads to increased classroom anxiety, which can interfere with students’ learning process, reduce their attention and memory, and even affect their learning motivation and strategy choices (Lavasani et al., 2011; Vogel and Schwabe, 2016). Therefore, this study proposes hypothesis H1: Academic procrastination can significantly predict the English classroom anxiety of non-English majors in the learning process.

### 1.2 The mediation of learning engagement between academic procrastination and English classroom anxiety

Learning engagement refers to a sustained, full-of-positive-emotion state that learners exhibit during the learning process, including factors such as the level of students’ engagement in learning tasks, active participation and concentration (Schaufeli et al., 2002). As a positive learning behavior practice, learning engagement can reflect the health of university students’ mental states (Cleofas, 2020). By cultivating university students’ learning engagement, they can build up positive learning attitudes and habits and lay a solid foundation for future development. When university students procrastinate on academic tasks, they may experience greater stress and anxiety because the accumulation of tasks can make them feel uncomfortable in class (Hong et al., 2015; Onwuegbuzie, 2004). In this case, learning engagement plays an important mediating role. It stimulates positive qualities such as hope, confidence and creativity in university students (Alvarez-Huerta et al., 2021; Reid and Solomonides, 2007; Zhong et al., 2021). When university students are highly engaged with and actively participating in learning tasks, they tend to be more motivated to complete the tasks and are better able to cope with underlying anxiety (Everingham et al., 2017). Therefore, this study proposes the hypothesis H2: Academic procrastination significantly predicts non-English majors’ English classroom anxiety during the learning process through the mediation of learning engagement.

### 1.3 The moderation of appraisals of intrinsic values between learning engagement and classroom anxiety

Appraisals of values refer to the value judgment of learner’s academic activities or outcomes, i.e., how learners perceive their own learning tasks and academic achievements (Pekrun, 2006). When university students truly identify with the importance of learning English and see it as an opportunity for self-development and growth, they are more likely to actively participate in their studies and make more efforts (Zarrinabadi and Rahimi, 2022). This learning engagement driven by intrinsic values pays more attention to the value of learning tasks and academic outcomes themselves, which can promote university students’ learning effectiveness and sense of achievement. Self-determination theory suggests that students who attach importance to their intrinsic self-value are more likely to focus on the task itself, have more courage in the face of challenges and are able to learn from the mistakes (Ryan and Deci, 2000, 2020). They are more willing to adopt active learning strategies, such as summarizing their knowledge of English, which helps to improve their understanding and application of the learning content. In contrast, students who lack intrinsic value may be more easily influenced by external evaluations and grades (Garaus et al., 2016; Husman and Lens, 1999). They are more concerned about the actual benefits of learning activities and outcomes, such as praise, rewards, which may affect their level of learning engagement. Therefore, this study proposes the hypothesis H3: Learning Engagement significantly predicts non-English majors’ English classroom anxiety during the learning process through the moderation of appraisals of intrinsic values.

### 1.4 The present study

**Figure 1:**
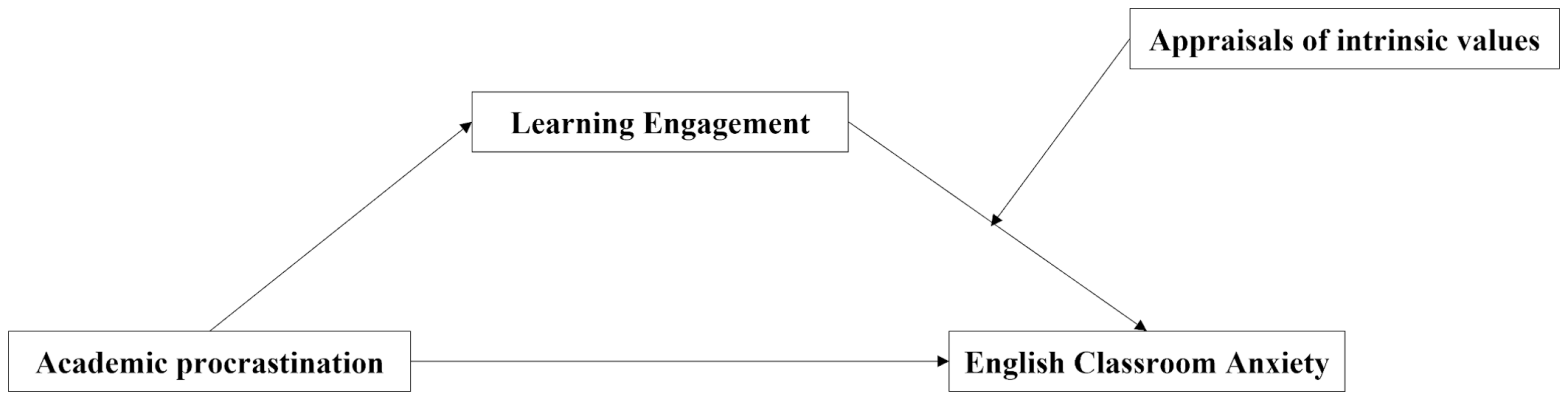
The proposed moderated mediation model.

The purpose of this study was to examine the relation mechanism between academic procrastination and English classroom anxiety among Chinese non-English majors. Specifically, we build a moderated mediation model to examine the following problems: (1) Does learning engagement mediate between academic procrastination and English classroom anxiety? (2) Do appraisals of intrinsic values moderate the relations between learning engagement and English classroom anxiety? (Figure 1).

## 2 Methods

### 2.1 Participants

In this study, a convenience sampling method was used to collect questionnaires from university students who were not majoring in English as a subject of study between January and February 2024.The sample was mainly from a public undergraduate college in Jiangsu Province. A ratio of 10: 1 between the number of subjects and the number of questionnaire questions is usually considered appropriate (Everitt, 1975; Nunnally and Bernstein, 1978). After deleting the invalid questionnaires such as those in which students fill out too quickly and those in which students answer randomly, a total of 1,079 valid questionnaires were recovered with the recovery rate being 89.92%. Wherein, there were 223 male (20.7%) and 856 female (79.3%). Among the places of student source, 521 (48.30%) students were from urban areas and 558 (51.70%) from rural areas. The age range of the subjects was 18–23 years old, with a mean age of 18.46 years old (SD = 0.65 years old). This study has been approved by the Science and Technology Ethics Committee of Nanjing Normal University of Special Education, ensuring that the study conforms to the ethical standards (NJTS20231221002).

### 2.2 Research tools

#### 2.2.1 Academic procrastination scale

The Procrastination Scale developed by Aitken was used in this study (Aitken, 1982). The scale consists of 19 items and is a one-factor structure. The Likert 5-point scoring method is adopted, ranging from 1 (not at all) to 5 (very much so). A higher score indicates more serious academic procrastination. The scale has been applied to Chinese student populations and has high reliability and validity (Geng et al., 2018). In the present study, the scale’s Cronbach’s α was 0.88.

#### 2.2.2 English classroom anxiety scale

Anxiety Scale for Foreign Language Classroom adapted by Jiang and Dewaele (2019) was adopted (Horwitz et al., 1986). The scale consists of a total of 8 items. The Likert 5-point scoring method is adopted, ranging from 1 (not at all) to 5 (very much so). A higher score indicates more serious English classroom anxiety. In the present study, the scale’s Cronbach’s *α* was 0.86.

#### 2.2.3 Learning engagement scale

The Learning Engagement Scale developed by Schaufeli et al. (2002) was chosen in this study. The scale consists of a total of 17 items, with three dimensions (Vigor, Dedication, Absorption). The Likert 7-point scoring method is adopted, ranging from 0 (never) to 6 (always). A higher score indicates higher level of learning engagement. In the present study, the scale’s Cronbach’s *α* was 0.97.

#### 2.2.4 Appraisals of intrinsic values scale

The Value Appraisals Scale adapted by Li was chosen in this study. Wherein, the fractal dimension of intrinsic value (Frenzel et al., 2007; Li, 2021) has been applied to Chinese university students’ English classroom teaching research, and has relatively high reliability and validity (Li, 2021). The dimension consists of a total of 5 items. The Likert 7-point scoring method is adopted, ranging from 1 (strongly disagree) to 7 (strongly agree). A higher score indicates higher intrinsic value of English learning. In the present study, the scale’s Cronbach’s *α* was 0.89.

### 2.3 Statistical analysis

Statistical analyses were performed using SPSS version 25.0. We performed descriptive statistics for all variables. We further tested the mediating role of learning engagement using the SPSS PROCESS macro (Model 4) (Hayes, 2017). Finally, we tested the moderating role of intrinsic value appraisal between learning input and English subject anxiety using the PROCESS macro (Model 14) (Hayes, 2017). Bias-corrected percentile Bootstrap methods were used to estimate 95% confidence intervals for the mediating effect. All analyses controlled for covariates.

## 3 Results

### 3.1 Common method deviation

The self-reported data of the participants are likely to have common method biases (Podsakoff et al., 2003). Therefore, Harman’s single-factor testing method was used to test common method deviation. The eigenvalues of 6 factors were greater than 1. The variation explained by the first common factor was 32.92%, less than the critical value of 40%. Therefore, there was no serious common method deviation in this study.

### 3.2 Descriptive statistics and correlations among variables

As shown in Table 1, there was a pairwise correlation between each other among university students’ academic procrastination, English classroom anxiety, learning engagement and appraisals of intrinsic values, with academic procrastination in significant positive correlation with English classroom anxiety, and in significant negative correlation with learning engagement and appraisals of intrinsic values; with English classroom anxiety in significant negative correlation with learning engagement and appraisals of intrinsic values; with learning engagement in significant positive correlation with appraisals of intrinsic values. Thus, H1 was supported.

**Table 1:** Descriptive statistics and correlations among variables (*N* = 1,079).

Variable	*M*	*SD*	1	2	3	4	5	6
1. Gender of university students	1.21	0.41	1					
2. English learning years	2.70	0.51	−0.13^**^	1				
3. Academic procrastination	48.23	10.36	0.12^**^	−0.12^**^	1			
4. English Classroom Anxiety	26.68	5.49	−0.06^*^	−0.08^*^	0.30^**^	1		
5. Learning Engagement	68.91	16.79	0.01	0.10^**^	−0.48^**^	−0.29^**^	1	
6. Appraisals of intrinsic values	24.47	5.76	−0.05	0.11^**^	−0.33^**^	−0.21^**^	0.58^**^	1

Gender of university students: 1 = female, 2 = male; English learning years: 1 = less than 6 years, 2 = 6–9 years, 3 = more than 9 years; *M* is mean value; SD is standard deviation. *p < 0.05, **p < 0.01, ***p < 0.001.

In addition, there were also significant correlations between each other among gender, academic procrastination and English classroom anxiety, and between each other among English learning years, academic procrastination, English classroom anxiety, learning engagement and appraisals of intrinsic values. Therefore, in the subsequent analysis they will be included as control variables in the model.

### 3.3 Testing for mediation effect

In order to explore the mediating effect of learning engagement between academic procrastination and Chinese university students’ English classroom anxiety, Hayes’s Model 4 of the PROCESS macro for SPSS was used to examine the mediating effect (Hayes, 2017). After controlling gender and English learning years, the results are shown in Table 2. The first step results indicate that academic procrastination significantly negatively predicts learning engagement (*β* = −0.48, *p* < 0.001). The second step results indicate that academic procrastination significantly positively predicts English classroom anxiety (*β* = 0.30, *p* < 0.001). The third step results indicate that learning engagement significantly negatively predicts English classroom anxiety (*β* = −0.18, *p* < 0.001), and the positive predicting function of academic procrastination is still significant to English classroom anxiety (*β* = 0.22, *p* < 0.001), which shows that learning engagement plays a mediating role between academic procrastination and English classroom anxiety. The bias-corrected percentile Boot-strap method was further adopted to examine indirect effects, with the mediation effect value being 0.09, SE being 0.02, 95%CI being [0.05,0.13], accounting for 28.63% of the total effect. Thus it can be seen that learning engagement has a significant partial mediating effect between academic procrastination and Chinese university students’ English classroom anxiety. Therefore, H2 was supported.

**Table 2:** Mediating effect analyses (*N =* 1,079).

Dependent variable	Independent variable	*R* ^2^	*F*	*β*	*SE*	*t*
Learning engagement	Gender of university students	0.23	109.00^***^	0.18	0.07	2.72^**^
	English learning years			0.10	0.05	1.83
	Academic procrastination			−0.48	0.03	−17.69^***^
English classroom anxiety	Gender of university students	0.10	39.86^***^	−0.26	0.07	−3.60^***^
	English learning years			−0.10	0.06	−1.79
	Academic procrastination			0.30	0.03	10.32^***^
English classroom anxiety	Gender of university students	0.13	38.44^***^	−0.23	0.07	−3.18^**^
	English learning years			−0.09	0.06	−1.50
	Academic procrastination			0.22	0.03	6.57^***^
	Learning engagement			−0.18	0.03	−5.56^***^

*p < 0.05, **p < 0.01, ***p < 0.001.

### 3.4 Test of the moderated mediation model

**Figure 2:**
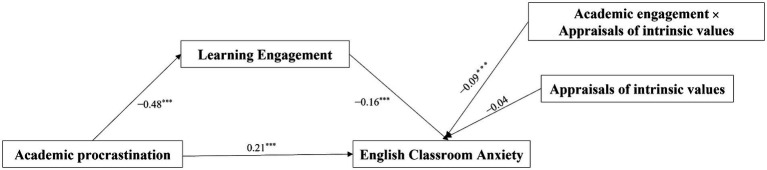
The moderated mediation model. *p < 0.05, **p < 0.01, ***p < 0.001.

Hayes’s Model 14 of the PROCESS macro for SPSS was used to examine the moderating mediation effect (Hayes, 2017). After controlling gender and English learning years, the results are shown in Figure 2. The interaction between learning engagement and appraisals of intrinsic values has a significant predictive effect on English classroom anxiety (*β* = −0.09, *p* < 0.001). Thus it can be seen that appraisals of intrinsic values has a significant moderating function between learning engagement and English classroom anxiety.

**Figure 3:**
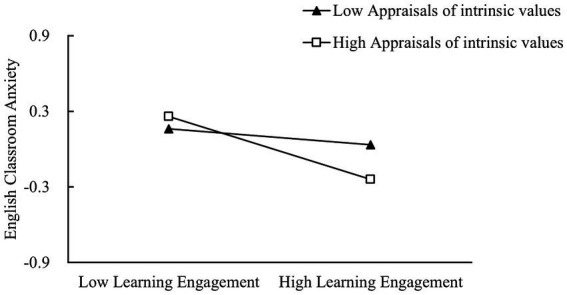
The moderating function of appraisals of intrinsic values between learning engagement and English classroom anxiety.

To further analyze the moderating effect of appraisals of intrinsic values between learning engagement and English classroom anxiety, participants were divided into two groups according to appraisals of intrinsic values scores: high appraisals of intrinsic values group (*M* + 1*SD*) and low appraisals of intrinsic values group (*M*-1*SD*), and the predictive effects of learning engagement on English classroom anxiety were investigated, respectively, in both groups. As shown in Figure 3, among university students with relatively higher appraisals of intrinsic values, learning engagement has a significant predictive effect on their English classroom anxiety (*β*_simple slope_ = −0.25, *p* < 0.001); for university students with relatively lower appraisals of intrinsic values, it is less predictive (*β*_simple slope_ = −0.06, *p* = 0.15). Therefore, H3 was supported.

## 4 Discussion

Academic procrastination and classroom anxiety have gradually gained attention in the field of education in recent years. Especially for English subject, as an international language, it is of great significance to university students’ academic development and personal growth. However, many university students are often faced with the difficulties of academic procrastination and classroom anxiety in learning English. Therefore, by exploring the mediating and moderating roles of Learning Engagement and intrinsic values between English learning procrastination and English classroom anxiety, teachers can better understand university students’ emotional state and adopt targeted and effective teaching strategies. This is of great significance to improve the English learning effect and academic level of university students.

### 4.1 The relation between academic procrastination and English classroom anxiety

The results show that non-English majors’ academic procrastination in learning can significantly positively predict English classroom anxiety. The higher academic procrastination is, the more severe the anxiety is, confirming hypothesis 1. This is similar to the results of previous studies (Wang, 2021; Zhang and Zhang, 2022). The results can also be explained by the “Appraisal-Anxiety-Avoidance” model of procrastination behavior (Milgram and Toubiana, 1999). The model suggests that the accumulation of academic skills will keep them in a state of constant rush, which may make college students confused and nervous in learning (Hong et al., 2015; Milgram and Tenne, 2000). When students realize that their academic achievement and performance are affected by procrastination, they may fall into feeling of self-reproach, helplessness and frustration (Jia et al., 2021). This negative emotion may manifest itself in the classroom and affect their positive attitudes and participation in learning (Albulescu et al., 2024). They may become reluctant to take the initiative to answer questions, participate in class discussions, or demonstrate their English skills, further deepening their anxiety (Azher et al., 2010; Matsuda and Gobel, 2004). Therefore, university administrators should ensure sufficient and reasonable allocation of teaching resources, such as providing diversified learning materials and online learning platforms, to meet the learning needs of different students and reduce the academic procrastination caused by insufficient resources. Teachers should also carry out mental health education activities regularly to detect and alleviate students’ anxiety in class. As for the college students themselves, they should make a reasonable study plan according to their own study situation, and avoid mental procrastination caused by confronting a large number of academic tasks at once.

### 4.2 The mediating role of learning engagement

The present study found that, learning engagement plays a partial mediating role between university students’ academic procrastination and English classroom anxiety, that is, university students’ academic procrastination affects English classroom anxiety through the mediating role of Learning Engagement, verifying the hypothesis 2. This study not only deepens our understanding of the causes of English classroom anxiety among non-English-major college students, but also further explores the mediating role of learning engagement in the relationship between academic procrastination and English classroom anxiety, providing us with a new dimension regarding the relationship between English learning and anxious psychological states. Specifically, it elucidates the important role of learning engagement in mitigating the negative effects of academic procrastination and reducing English classroom anxiety, providing valuable insights into enhancing students’ learning experience and psychological well-being. In addition to the overall mediating effect, each individual aspect of the mediating process deserves attention.

In response to the phenomenon of academic procrastination, the present study found a negative correlation between it and learning engagement, a finding that is consistent with the results of several previous studies (Closson and Boutilier, 2017; Li et al., 2023). The findings support the Resource Conservation Theory, a model that suggests that the process of learning engagement is itself a process of resource accumulation (Alarcon et al., 2011; Liu et al., 2023). Students continue to construct knowledge frameworks and enhance language skills in the process of active learning, and these resources become a solid backbone for them to cope with learning challenges and mitigate academic procrastination (Martínez et al., 2019). Specifically, students who experience frequent academic procrastination behaviors may reduce their effective investment time in English language learning, such as pre-study, revision and motivation to participate in English classroom activities. For Learning Engagement and English classroom anxiety, the results of this study showed a negative correlation. This is similar to previous studies (Everingham et al., 2017; Wang et al., 2023). This may be due to the fact that when students actively invest time in acquiring English language knowledge, they tend to be able to manage tensions and reduce anxiety in the English classroom environment more effectively. This phenomenon is also consistent with the affective filtering hypothesis, which states that affective factors play an important role in the process of learning a second language (Li and Zhou, 2023). If college students are engaged in the English learning process, the emotional filter will be in a more relaxed state, which is conducive to better mastery of English knowledge as well as reducing anxiety in the English classroom. Therefore, college administrators need to create a positive and supportive learning environment and encourage students to participate in learning activities, for example, by organizing a variety of English academic lectures to stimulate students’ interest in learning and promote their engagement in the English learning process. At the same time, teachers should pay attention to students’ emotional changes, especially anxiety in the English classroom. Through positive feedback and patient guidance, they can help students build up their self-confidence and reduce the anxiety caused by language barriers or learning pressure. For college students themselves, they should take the initiative to set clear learning goals, actively participate in classroom interactions, and continuously improve their language proficiency and sense of learning efficacy.

### 4.3 The moderating role of appraisals of intrinsic values

The present study found that, appraisals of intrinsic values played a moderating role in indirect effects between learning engagement and English classroom anxiety, verifying the hypothesis 3. Appraisals of intrinsic values not only positively predicted students’ learning engagement, but also moderated the effect of learning engagement on English classroom anxiety. Specifically, the negative association between Learning Engagement and English classroom anxiety was relatively weak for those college students with higher appraisals of intrinsic values. This result supports the control-one-value theory, which suggests that appraisals of intrinsic values is an important factor in arousing academic emotions and enhances learning engagement and attractiveness to learners (González et al., 2016; Pekrun, 2006). When college students perceive an English learning task as highly valuable for their personal growth or intrinsic needs, they are more likely to remain highly engaged in learning and experience less anxiety as a result (Wang et al., 2017). However, university students with lower appraisals of intrinsic values may have difficulty experiencing a sense of achievement and satisfaction in the English learning process. As a result they may be less engaged in learning and more vulnerable to the negative effects of English classroom anxiety. This result is also consistent with self-determination theory, which suggests that an individual’s intrinsic motivation and satisfaction with an activity are important factors that drive their behavior (Ryan and Deci, 2000). Students with a high intrinsic value assessment are more likely to experience the enjoyment of learning English, which leads to increased engagement in learning and self-efficacy, and ultimately lower levels of anxiety (Ma et al., 2018). Therefore, university administrators should help students recognize the importance of English language learning for personal growth, intercultural communication and future career development. When students recognize the intrinsic value of English language learning, they are more likely to maintain a sustained enthusiasm for learning. In addition, teachers should guide students to see learning English as a process of exploring the world and broadening their horizons, rather than simply a task to pass a test. When students see the positive impact of language learning on their personal development, they will feel less anxious about the process of learning English. For the students themselves, they need to think deeply about their motivation for learning English. This helps to shift motivation from external pressures such as exams and parental expectations to intrinsic values such as personal interest and cultural exploration of the outside world.

### 4.4 Limitations

There are also some shortcomings in this study. First, although the cross-sectional in-depth research in this study is on the basis of theoretical and empirical research, there are still some methodological limitations. To further verify the causal relation, future studies can use experimental designs to control other variables and observe the effects of specific interventions. Secondly, this study does not collect data on both teachers and students in English class. Therefore, in future research, both teachers and students can be investigated to better understand the interaction and teaching effect in English class. Finally, this study adopted convenience sampling with the sample coming from a normal university. However, in the context of Chinese culture, these majors are more favored by women. Future research should be devoted to expanding the scope of sampling, and by covering groups of subjects of different ages, different educational backgrounds, and different learning environments, it can more comprehensively reveal the influencing factors and changing patterns of English classroom anxiety.

### 4.5 Practical implications

Despite these limitations, our study demonstrates significant value at both the theoretical and practical levels. First, this study provides insights into the mediating variable of learning engagement and enriches the theoretical framework between procrastination and English classroom anxiety. Second, our study further reveals the moderating role of intrinsic value appraisal. When college students learn English out of intrinsic values such as interest, personal growth, or future planning, they can effectively reduce English classroom anxiety. Therefore, educators can help college students establish positive learning attitudes by enhancing their intrinsic value of English learning, which can reduce procrastination behavior and classroom anxiety.

## 5 Conclusion

(1) Non-English majors’ academic procrastination has a significant negative predictive effect on English classroom anxiety. (2) Learning engagement plays a partial mediating role between non-English majors’ academic procrastination and English classroom anxiety. (3) The latter half of the way in which learning engagement mediates between academic procrastination and English classroom anxiety is moderated by appraisals of intrinsic values. Specifically, under the high appraisals of intrinsic values, the impact of non-English majors’ Learning Engagement on English classroom anxiety is significantly enhanced.

## Data Availability

The original contributions presented in the study are included in the article/supplementary material, further inquiries can be directed to the corresponding author.
